# Feline vector-borne pathogens in the north and centre of Portugal

**DOI:** 10.1186/1756-3305-6-99

**Published:** 2013-04-15

**Authors:** Hugo Vilhena, Verónica L Martinez-Díaz, Luís Cardoso, Lisete Vieira, Laura Altet, Olga Francino, Josep Pastor, Ana C Silvestre-Ferreira

**Affiliations:** 1 Department of Veterinary Medicine, Escola Universitária Vasco da Gama, Coimbra, Portugal; 2 Baixo Vouga Veterinary Hospital, Águeda, Portugal; 3 Department of Animal and Food Sciences, Agrigenomics Research Centre (CRAG), Veterinary Faculty, Universitat Autònoma de Barcelona, Bellaterra (Barcelona), Spain; 4 Department of Veterinary Sciences, School of Agrarian and Veterinary Sciences, Universidade de Trás-os-Montes e Alto Douro, Vila Real, Portugal; 5 Parasite Disease Group, Instituto de Biologia Molecular e Celular (IBMC), Universidade do Porto, Oporto, Portugal; 6 Veterinary Service for Molecular Genetics, Department of Animal and Food Sciences, Veterinary Faculty, Universitat Autònoma de Barcelona, Bellaterra (Barcelona), Spain; 7 Department of Animal Medicine and Surgery, Veterinary Faculty, Universitat Autònoma de Barcelona, Bellaterra (Barcelona), Spain

**Keywords:** Cats, Feline vector-borne diseases, PCR, Portugal

## Abstract

**Background:**

In recent years, several clinical cases and epidemiological studies of feline vector-borne diseases (FVBD) have been reported worldwide. Nonetheless, information on FVBD agents and their prevalence in Portugal is scarce.

**Methods:**

Three-hundred and twenty domestic cats presented to 30 veterinary medical centres in the north and centre regions of Portugal were randomly sampled. Blood was assayed by real-time polymerase chain reaction (PCR) for genera *Anaplasma*/*Ehrlichia*, genus *Babesia*, *Hepatozoon canis*, *Hepatozoon felis*, *Leishmania infantum* and the genus *Rickettsia*. *Babesia*-positive samples were further tested for *Babesia canis* and *Babesia vogeli*.

**Results:**

Eighty (25.0%) out of the 320 cats were positive to at least one vector-borne agent, including seven (2.2%) cats co-infected with two agents. Two cats (0.6%) were infected with *Anaplasma*/*Ehrlichia* spp., four (1.3%) with *B. canis*, 26 (8.1%) with *B. vogeli*, 50 (15.6%) with *H. felis*, one (0.3%) with *L. infantum* and four (1.3%) with *Rickettsia* spp. No cat tested positive for *H.* canis. One cat (0.3%) was co-infected with *B. canis* and *B. vogeli*, three (0.9%) with *B. vogeli* and *H. felis*, one (0.3%) with *H. felis* and *L. infantum*, and two (0.6%) with *H. felis* and *Rickettsia* spp.

**Conclusions:**

A considerable prevalence of infection with vector-borne pathogens among the domestic feline population of the north and centre of Portugal has been revealed by the present study. Additionally, this is the first detection of *B. vogeli* in cats from Europe and of *H. felis* in cats from Portugal.

## Background

Vector-borne diseases compromise a variety of infectious illnesses caused by several agents, including viruses, bacteria, protozoa and helminthes, which are transmitted by ticks, fleas, mosquitoes and phlebotomine sand flies [1,2]. Many of these agents are emerging or re-emerging pathogens [3] and some of them are of zoonotic concern [4,5]. The frequency of some vector-borne diseases is increasing in Europe, partially due to climatic alterations that have a direct impact on the abundance, geographical distribution and vectorial capacity of arthropod vectors, but also due to the increased mobility of human beings and animals, which further promote the circulation and exchange of vectors and infectious agents [1,3,6,7].

Although several vector-borne agents cause morbidity and mortality in the domestic feline population [8], the importance of some of them as a cause of disease has not yet been clearly determined [9]. This lack of knowledge, associated with the unawareness of the distribution and ecology of feline vector-borne diseases (FVBD) of zoonotic concern, has impaired the implementation of effective control measures to prevent infection of cats, other animals and human beings [1].

The diagnosis of infectious diseases in cats may be challenging, as some infections can occur in healthy cats [10], and whenever present clinical signs are frequently non-specific [1,11]. Most of the agents are often present in low numbers in peripheral blood, are difficult to cultivate *in vitro*, elicited specific antibody responses may be inconsistent [11], and serological cross-reactivity can exist between some organisms [12]. Many problems of serology are circumvented by the use of the polymerase chain reaction (PCR) [12,13]. In addition to accurate detection of infectious agents in animals, human beings and arthropod vectors, DNA-based techniques allow species characterization of different pathogens, which can be important for treatment and prognosis [6]. Compared with the conventional method, real-time PCR can have a higher sensitivity in some diseases [14] and is a useful tool both for diagnosis and treatment monitoring [13].

Previous clinical case reports [15], and serological or molecular surveys [16-19] have described infection with different vector-borne organisms in cats from Portugal. Nonetheless, information about agents of FVBD and their prevalence in Portugal is scarce. The aims of the present study were to identify the presence and prevalence of vector-borne agents from genera *Anaplasma*, *Babesia*, *Ehrlichia*, *Hepatozoon*, *Leishmania* and *Rickettsia* in cats from the north and centre regions of Portugal, by means of real-time PCR, and to identify risk factors associated with infection.

## Methods

### Cats and samples

Three-hundred and twenty domestic cats from the north (n = 140) and centre (n = 180) of Portugal were randomly sampled in 30 veterinary medical centres, without inclusion/exclusion criteria or pre-established minima/maxima. The number of cats sampled per centre ranged from 1 to 58. This study was ethically approved by the board of the University of Trás-os-Montes e Alto Douro veterinary teaching hospital as complying with the Portuguese legislation for the protection of animals (Law no. 92/1995, from September the 12th).

Whole blood samples were obtained by jugular or cephalic venipuncture into EDTA tubes and stored at −20°C until DNA extraction. Whenever available, medical and geographic data of each cat was collected, including gender, age, breed, living conditions, clinical status, and feline immunodeficiency virus (FIV)/feline leukaemia virus (FeLV) infections status (Table 1). Practitioners classified the cats as clinically suspect, if they had clinical signs compatible with a FVBD, or non-suspect, when they were apparently healthy or had clinical signs not compatible with an infectious disease. Compatible physical signs and clinicopathological abnormalities comprised anorexia, weight loss, gastrointestinal alterations, anemia, thrombocytopenia, leukocytosis or leukopenia, jaundice and dermatological or ocular manifestations without any other attributable aetiology.

**Table 1:** Prevalence of infection with vector-borne pathogens in cats from the north and centre of Portugal, as determined by PCR

**Independent variable/category**	**No. of cats tested (%)**	**No. of positive cats (%)**						
		***Anaplasma*/*Ehrlichia***	***B. canis***	***B. vogeli***	***H. felis***	***L. infantum***	***Rickettsia***	**≥1 agent(s)**
Gender	320							
Female	142 (44.4)	2 (1.4)	2 (1.4)	14 (9.9)	19 (13.4)	1 (0.7)	0 (0.0)	36 (25.4)
Male	178 (55.6)	0 (0.0)	2 (1.1)	12 (6.7)	31 (17.4)	0 (0.0)	4 (2.2)	44 (24.7)
Age (years)	312							
[0.4-1.5]	90 (28.8)	0 (0.0)	2 (2.2)	11 (12.2)^a^	13 (14.4)	0 (0.0)	0 (0.0)	23 (25.6)
[2.0-6]	157 (50.3)	1 (0.6)	2 (1.3)	13 (8.3)	25 (15.9)	1 (0.6)	3 (1.9)	42 (26.8)
[7-20]	65 (20.8)	1 (0.6)	0 (0.0)	1 (1.5)^a^	11 (16.9)	0 (0.0)	1 (0.6)	14 (21.5)
Breed	320							
DSH	274 (85.6)	2 (0.7)	4 (1.5)	19 (6.9)	43 (15.7)	1 (0.4)	3 (1.1)	66 (24.1)
Pure breed	46 (14.4)	0 (0.0)	0 (0.0)	7 (15.2)	7 (15.2)	0 (0.0)	1 (2.2)	14 (30.4)
Housing	316							
Totally indoors	124 (39.2)	0 (0.0)	0 (0.0)	7 (5.6)	20 (16.1)	0 (0.0)	2 (1.6)	26 (21.0)
Outdoors access	192 (60.8)	2 (1.0)	4 (2.1)	19 (9.9)	30 (15.6)	1 (0.5)	2 (1.0)	54 (28.1)
FeLV	117							
Negative	107 (91.5)	0 (0.0)	1 (0.9)	9 (8.4)	15 (14.0)	0 (0.0)	2 (1.9)	24 (22.4)
Positive	10 (8.5)	0 (0.0)	0 (0.0)	1 (10.0)	0 (0.0)	0 (0.0)	0 (0.0)	1 (10.0)
FIV	117							
Negative	97 (82.9)	0 (0.0)	1 (1.0)	10 (10.3)	13 (13.4)	0 (0.0)	2 (2.1)	23 (23.7)
Positive	20 (17.1)	0 (0.0)	0 (0.0)	0 (0.0)	2 (10.0)	0 (0.0)	0 (0.0)	2 (10.0)
Clinical status	300							
Non-suspect	132 (44.0)	2 (1.5)	2 (1.5)	16 (12.1)	17 (12.9)	1 (0.8)	1 (0.8)	34 (25.8)
Suspect	168 (56.0)	0 (0.0)	2 (1.2)	10 (6.0)	33 (19.6)	0 (0.0)	3 (1.8)	46 (27.4)
Total	320 (100)	2 (0.6)	4 (1.3)	26 (8.1)	50 (15.6)	1 (0.3)	4 (1.3)	80 (25.0)

^a^*p* = 0.031; *B. canis*: *Babesia canis*; *B. vogeli*: *Babesia vogeli*; DSH: domestic short-haired; FeLV: feline leukaemia virus; FIV: feline immunodeficiency virus; *H. felis*: *Hepatozoon felis*; *L. infantum*: *Leishmania infantum*; PCR: polymerase chain reaction.

### DNA extraction, PCR amplification and sequencing

DNA was obtained from 0.5 ml of peripheral blood, as previously described [14].

Real-time quantitative (q) PCR for *Leishmania infantum* was carried out according to the method described by Francino *et al*. [14]. The targets of primers (Table 2) and TaqMan-MGB probes were conserved regions of the kinetoplastic minicircle of *L. infantum*.

**Table 2:** Primer sequences of the tested vector-borne pathogens (genera or species)

**Agents**	**Amplified region**	**Primer forward (5′-3′)**	**Primer reverse (5′-3′)**	**Final [primer] (μM)**
*Anaplasma/Ehrlichia* spp.	16S rRNA	GGGTGAGTAATGCRTAGGAATCTACCTAGTA	GGATTATACAGTATTACCCAYCATTTCTARTG	0.5
*Babesia* spp.	18S rRNA	GTGGCTTTTCCGATTCGTCG	TTCCTTTAAGTGATAAGGTTCACAAAACTT	0.3
*Babesia canis*	18S rRNA	CGGTTTGACCATTTGGTTGGTTA	CCATGCTGAAGTATTCAAGACAAAAGT	0.3
*Babesia vogeli*	18S rRNA	CATTCGTTTGGCTTTTTCGAG	CCATGCTGAAGTATTCAAGACAAAAGT	0.3
*Hepatozoon felis*	18S rRNA	CTTACCGTGGCAGTGACGGT	TGTTATTTCTTGTCACTACCTCTCTTATGC	0.3
*Leishmania infantum*	kinetoplast DNA	AACTTTTCTGGTCCTCCGGGTAG	ACCCCCAGTTTCCCGCC	0.9
*Rickettsia* spp.	16S rRNA	AGCCTGATCCAGCAATACCGA	CGGGGCTTTTTCTGCAAGTAA	0.3

For the other agents, samples were submitted to different qPCR for genera *Anaplasma*/*Ehrlichia*, genus *Babesia*, *Hepatozoon canis*, *Hepatozoon felis* and genus *Rickettsia*. *Babesia-*positive samples were further tested for *Babesia canis* and *Babesia vogeli*. All primers were designed by The Molecular Genetics Veterinary Service, (Universitat Autònoma de Barcelona) (Table 2). Specificity of each primer was: (i) tested *in silico*, to avoid cross-amplification with other FVBD agents, using sequence information available in Genbank and RDP II databases; and (ii) also validated by the amplification of the positive control for which the PCR had been designed and by the absence of amplification in samples positive to other pathogens; and (iii) by DNA sequencing of some positive samples to confirm agents *B. canis* (n = 1), *B. vogeli* (n = 3) and *H. felis* (n = 4).

For all agents, qPCR amplification was carried out in a final volume of 20 μl using FastStart Universal SYBR Green Master (Roche), 4 μl of diluted DNA and a final primer concentration depending on the amplified pathogen (Table 2). Thermal cycling profile was 50°C for 2 min and 95°C for 10 min, followed by 40 cycles at 95°C for 15 s and 60°C for 1 min. Specificity assessment of qPCR was performed by adding a dissociation curve analysis at the end of the run. The internal reference for cat genomic DNA was the eukaryotic 18S RNA Pre-Developed TaqMan Assay Reagents (Applied Byosystems), which ensured proper qPCR amplification of each sample and that negative results corresponded to true negative samples rather than to a problem with DNA loading, sample degradation or PCR inhibition. Positive qPCR controls were obtained from clinical samples previously amplified and sequenced to confirm the pathogen. Water was used as a negative control. The product of the real-time PCR was sequenced with the BigDye Terminator Cycle Sequencing Ready Reaction Kit (AB, Live Technologies) using the same primers. Sequences obtained were compared with GenBank database (http://www.ncbi.nlm.nih.gov/BLAST).

### Statistical analysis

Prevalences of infection relative to the independent variables (i.e. gender, age, breed, housing conditions, FeLV/FIV infections and clinical status) were compared by Chi-square or Fisher’s exact tests. A *p* value < 0.05 was considered as statistically significant. Analyses were performed with SPSS® 11.5 software for Windows (SPSS Inc).

## Results

Table 1 displays data on the prevalence of infection with vector-borne agents among the 320 cats assessed in this study. Cats (n = 312) were aged from 5 months to 20 years (mean: 4.32 years ± 3.88 standard deviation). Pure breed cats were mainly Persians and Siamese. Absolute numbers and proportions (positive/tested cats) of single and co-infections are shown in Table 3.

**Table 3:** Prevalence of single and co-infections with vector-borne pathogens in 320 cats from north and centre Portugal, as determined by PCR

**Agent(s)**	**No. of positive cats**	**%**
Single infections	73	22.8
*Anaplasma* spp./*Ehrlichia* spp.	2	0.6
*B. canis*	3	0.9
*B. vogeli*	22	6.9
*H. felis*	44	13.8
*L. infantum*	0	0.0
*Rickettsia* spp.	2	0.6
Co-infections	7	2.2
*B. canis* + *B. vogeli*	1	0.3
*B. vogeli* + *H. felis*	3	0.9
*H. felis* + *L. infantum*	1	0.3
*H. felis* + *Rickettsia* spp.	2	0.6
Total	80	25.0

*B. canis*: *Babesia canis*; *B. vogeli*: *Babesia vogeli*; *H. felis*: *Hepatozoon felis*; *L. infantum*: *Leishmania infantum*; PCR: polymerase chain reaction.

Eighty cats (25.0%) were qPCR positive to at least one of the tested agents, including seven (2.2%) cats co-infected with two agents. Two cats (0.6%) were infected with *Anaplasma*/*Ehrlichia* spp., four (1.3%) with *B. canis*, 26 (8.1%) with *B. vogeli*, 50 (15.6%) with *H. felis*, one (0.3%) with *L. infantum* and four (1.3%) with *Rickettsia* spp. No cat tested positive to *H. canis*. One cat (0.3%) was co-infected with *B. canis* and *B. vogeli*, three (0.9%) with *B. vogeli* and *H. felis*, one (0.3%) with *H. felis* and *L. infantum*, and two (0.6%) with *H. felis* and *Rickettsia* spp.

Sequencing confirmed *B. canis* in the one cat (100% relatedness to GenBank HQ662634.1), *B. vogeli* in the three cats (100% relatedness to GenBank JX871885.1) and *H. felis* in the four cats (100% relatedness to GenBank JQ867388.1) whose PCR products were sequenced. Because of the small quantities of amplified bacterial DNA in the genus-specific PCR, additional species-specific PCR assays or DNA sequencing were not performed for those samples positive to genera *Anaplasma*/*Ehrlichia* and *Rickettsia*.

A statistically significant difference (*p* = 0.031) was found between the prevalence of infection with *B. vogeli* in cats aged 5–18 months (0.42-1.5 years) and in cats aged 7–20 years old (12.2% versus 1.5%, respectively) (Table 1). No other significant differences were found for the prevalence of infection with each vector-borne agent, at least one agent (≥ 1 agent[s]) or co-infections (2 agents), regarding all the independent variables.

## Discussion

The present study represents the most comprehensive investigation on FVBD performed in Portugal, in terms of the number of tested cats and extension of the covered geographical area, and reveals a considerable prevalence of infection in domestic cats from the north and centre regions of Portugal. *Anaplasma/Ehrlichia* spp., *B. canis*, *B. vogeli*, *H. felis*, *L. infantum* and *Rickettsia* spp. were detected among the assayed feline population.

Several ehrlichial and rickettsial infections are shared by man and companion animals [20]. In the present study, *Anaplasma*/*Ehrlichia* spp. and *Rickettsia* spp. DNA were detected in 0.6% and 1.3% of the cats, respectively. In cats from southern Portugal, seroprevalences (by immunofluorescence antibody tests) were 13.5% for *Anaplasma phagocytophilum* and 18.9% for *Rickettsia conorii*[17], and 26.3% for *R. conorii*/*Rickettsia felis*[19]. Furthermore, Breu *et al*. [19] also reported feline infection by *Ehrlichia canis* in Portugal. A national serological study on canine vector-borne diseases in Portugal detected a significantly higher seroprevalence of antibodies to *Anaplasma* spp. and *E. canis* in dogs from southern Portugal, when compared to dogs from the northern and central regions [7].

The present study represents the first report on the prevalence of *Babesia* spp. in cats from Portugal. A higher prevalence of *Babesia* spp. was found in Portuguese cats (9.4%), in comparison with that detected in cats from Barcelona, Spain (0/100) by Tabar *et al.*[12]. Interestingly, it can also be presumed that the most prevalent piroplasm in the Portuguese feline population is *B. vogeli*, instead of *B. canis*, which was the piroplasm most frequently detected in dogs with babesiosis from the north of Portugal [21]. So far, feline infection with *B. vogeli* has only been described in cats from Trinidad, Trinidad and Tobago [22], and Bangkok, Thailand [23]. To the best of our knowledge, this is the first time that infection with *B. vogeli* has been detected in cats from Europe.

Feline co-infections with other erythrocytic pathogens such as *Mycoplasma* spp., *Cytauxzoon felis* or other species of *Babesia* may be possible [24,25]. In the present study, only one cat was found co-infected with *B. canis* and *B. vogeli* out of the 29 cats infected with *Babesia* spp. Infection with *B. canis* and/or the *Babesia microti*-like piroplasm (syn. *Theileria annae*) was previously described in three cats from Portugal [26], but no information is available on the geographical origin of those cats. The *B. microti*-like piroplasm has also recently been found in dogs from northern Portugal affected by babesiosis [27]. In the present study, other species of the genera *Babesia* and *Theileria* were not assessed. Nevertheless, as the entire results positive to the genus *Babesia* had an assigned species (i.e. *B. vogeli*, *B. canis* or both), although not impossible, a co-infection with the *B. microti*-like piroplasm seems unlikely.

Age has been described as a predisposing factor for feline infection with *Babesia* spp., with younger cats (less than 3 years old) more predisposed to infection in endemic areas [24,28], and older cats more susceptible to the disease following relocation to an endemic area or in conjunction with concurrent disease, immunosuppression or severe trauma [24]. In the present study, juvenile cats (≤ 1.5 year) had a significantly higher prevalence of *B. vogeli* infection in comparison with geriatric cats (≥ 7 years), probably because of the less mature immune status of young cats.

Infection with *Hepatozoon* spp. is frequently reported in dogs [29] but not in cats. Furthermore, the *Hepatozoon* species that infect cats have not been definitely characterized [30,31]. Some authors have suggested that *H. canis* is the agent responsible for feline infection, but a new, yet unnamed, species of *Hepatozoon* genetically distinct from *H. canis* was recently detected in cats from southern and northeastern Spain [30,32]. Infection with *H. felis* was firstly described by Tabar *et al.* in cats from Barcelona [12].

This is also the first report of feline infection with *H. felis* in Portugal. Molecularly confirmed infection with *H. canis* in cats from southern Portugal [19] and in a dog from northern Portugal [21] had already been described. The detected 15.6% prevalence of infection with *H. felis* in the present study is similar to that of *Hepatozoon* spp. (16%) described by Ortuño *et al.*[30] in stray cats from Barcelona (*p* = 0.960), but higher than the 0.6% of *Hepatozoon* spp. found in Spanish domestic cats from a non-identified geographical background [32] and the 4.0% of *H. felis* in domestic cats from Barcelona [12] (*p* < 0.001 and *p* = 0.002, respectively). Moreover, the present study sustains the fact that *Hepatozoon* infection is widespread in the feline population of the Iberian Peninsula. Baneth *et al.*[33], in a study from Israel, detected that most infected cats were young domestic short-haired males and that there was an over-representation of cats with retroviral disease. In the present study, no statistically significant association was found between infection with *H. felis* and independent variables including clinical status and FIV/FeLV infection (Table 1).

Leishmaniasis is an endemic zoonosis prevalent in the Mediterranean basin [34,35]. The increase in the number of infections and disease cases reported in recent years, together with the results described in different prevalence studies, suggest that cats can act as a secondary reservoir host instead of an accidental one in areas where *Leishmania* spp. are endemic [36-38]. Several surveys of *Leishmania* spp. infection in cats have been performed in different countries by different techniques, with prevalences ranging between 0% and 68% [9,16,18,39-43]. In the present study, detected prevalence (0.3%) might have been different if the qPCR was carried out with another tissue sample, such as bone marrow, spleen or liver. Results are lower than the 2.8% seroprevalence (by the direct agglutination test and an enzyme-linked immunosorbent assay) found in cats from northern Portugal [18] and also lower than the 20.3% obtained in blood samples from cats of Greater Lisbon (southern-central Portugal) by PCR [16] (*p* = 0.01 and *p* < 0.001, respectively). For the appropriate agents under assessment in the present study, comparative differences in the prevalence values can be related to different detection techniques (serology versus molecular analysis) as well as to a different geographical origin of cats (north and centre versus south). The latter may determine differences in climatic conditions, arthropod vector survival and agent transmission rate.

Associations between housing conditions and prevalence of infection were not found among cats for any one of the agents. Conversely, in a comparable study on canine vector-borne diseases, also in Portugal, a significantly higher sero-positivity to at least one agent (i.e. *Dirofilaria immitis*, *E. canis*, *Borrelia burgdorferi* sensu lato, *Anaplasma* spp. and/or *L. infantum*) has been found in clinically suspect dogs with an outdoor or mixed lifestyle [7].

## Conclusions

In the north and centre regions of Portugal, a high prevalence of infection with *B. vogeli* and *H. felis*, and a relatively low prevalence of infection with *Anaplasma*/*Ehrlichia* spp., *B. canis*, *L. infantum* and *Rickettsia* spp. were found in cats. Further studies on these and other vector-borne agents are needed to better understand their epidemiological and clinical importance. It is also necessary to call on veterinarians and owners to adopt effective control measures, including chemoprophylaxis against the ectoparasite vectors, in order to prevent infection of cats with agents of FVBD and their potential transmission to other domestic and wild animals as well as to human beings.

## Competing interests

The authors declare that they have no competing interests.

## Authors’ contributions

HV collected samples and clinical data, performed data analysis and drafted the manuscript; VLM-D performed DNA extraction and molecular analyses; LC co-supervised the study, performed data analysis and revised the manuscript; LV collected samples and clinical data; LA and OF designed the primers, supervised molecular analyses and provided conceptual advice; JP and ACS-F planned and supervised the study, coordinated sample collection and reviewed the manuscript. All authors read and approved the final manuscript.
